# Communication in cancer genetic counselling: does it reflect counselees' previsit needs and preferences?

**DOI:** 10.1038/sj.bjc.6602570

**Published:** 2005-04-19

**Authors:** A H Pieterse, A M van Dulmen, M G E M Ausems, F A Beemer, J M Bensing

**Affiliations:** 1 NIVEL (Netherlands Institute for Health Services Research), PO Box 1568, 3500 BN Utrecht, the Netherlands; 2 Department of Medical Genetics, University Medical Centre Utrecht, PO Box 85090, 3508 AB Utrecht, the Netherlands; 3 Department of Health Psychology, Utrecht University, PO Box 80125, 3508 TC Utrecht, the Netherlands

**Keywords:** cancer genetics, precounselling needs, communication

## Abstract

This study sought to describe counsellor–counselee interaction during initial cancer genetic counselling consultations and to examine whether the communication reflects counselees' previsit needs. A total of 130 consecutive counselees, referred mainly for breast or colon cancer, completed a questionnaire before their first appointment at a genetic clinic. Their visit was videotaped. Counselee and counsellor verbal communications were analysed and initiative to discuss 11 genetics-specific conversational topics was assessed. The content of the visit appeared relatively standard. Overall, counselees had a stronger psychosocial focus than counsellors. Counsellors directed the communication more and initiated the discussion of most of the topics assessed. Counselees did not appear to communicate readily in a manner that reflected their previsit needs. Counsellors provided more psychosocial information to counselees in higher need for emotional support, yet did not enquire more about counselees' specific concerns. New counselees may be helped by receiving more information on the counselling procedure prior to their visit, and may be advised to prepare the visit more thoroughly so as to help them verbalise more their queries during the visit.

The exchange of information is the core of cancer genetic counselling. Preferably, it supports a ‘caring, professional relationship that offers guidance, but allows individuals and families to come to their own decisions’ as put forward by the Proposed international guidelines on ethical issues in medical genetics and genetic services (1998). Several authors argued that counsellors should try to elicit counselees' needs and preferences at the outset, and to adapt their counselling accordingly (Michie *et al*, 1998; Bernhardt *et al*, 2000; Lobb *et al*, 2001). Findings on communication within general practice (Marvel *et al*, 1999; Street, 1991) and oncology (Ford *et al*, 1996) suggest that physicians rarely solicit patients' concerns and questions. At the same time, studies revealed that patients' strong desire for medical information did not prompt them to engage frequently in information-seeking behaviours (Beisecker and Beisecker, 1990). Butow and Lobb (2004) described the typical process and content of first genetic counselling consultations with women from high-risk breast cancer families. They found that counsellors provided detailed information on various aspects related to familial breast cancer but did not consistently elicit emotional concerns. Indeed, in another analysis of the data, Lobb *et al* (2002) found that counsellors did not adapt their counselling according to women's expectations or anxiety. Rather, women's professional occupation and disease status were predictive of the type of information that was provided and how this was delivered.

The purpose of the present study was two-fold. First, it aimed to describe counsellor–counselee interaction during initial cancer genetic counselling consultations in terms of affective and task-oriented exchange and topics discussed. Second, it aimed to examine whether counselee–counsellor communication reflects counselees' previsit needs, as measured using the QUOTE-gene^ca^ (Pieterse *et al*, 2005). This counselee-centred instrument revealed four generic needs related to informational and psychosocial issues, and four cancer-specific needs related to receiving explanations about medical and emotional issues specifically. Regarding counselee communication, it was hypothesised that counselees with higher prior informational needs would ask more medical and psychosocial questions, and that counselees with higher prior emotional needs would provide more psychosocial information and show more concern. It was further expected that counselees considering it important to be taken seriously and be listened to would offer more information on their reasons for attending. With regard to counsellor communication, it was expected that counsellors would pay more attention to counselees' needs by showing more empathy, inquiring more about psychosocial issues, and providing more psychosocial and medical information and counselling, with counselees with higher prior needs relating to these aspects.

## MATERIALS AND METHODS

### Participants

#### Counselees

Participating counselees were recruited from the consecutive new referrals for cancer genetic counselling at the Department of Medical Genetics of the University Medical Centre Utrecht, the Netherlands, between March 2001 and August 2003. Inclusion criteria were aged 18 years or older and being the first in the family to seek genetic counselling.

#### Counsellors

All clinical geneticists, residents in clinical genetics (of whom two finished their training during the study period), and genetic nurses (of whom four finished their training) providing cancer genetic counselling agreed to participate in the study. All will be referred to as ‘counsellor’.

### Procedure

The Medical Ethical Committee of the university hospital granted permission to conduct the study, including videorecording of the consultations. Participants were sent a questionnaire within a week before their first consultation and were asked to complete it before their visit at the clinic, along with an informed consent form. At the start of the consultation, after collecting the questionnaire and the signed informed consent form, the counsellor notified the counselee about the fact that the video had started recording. The unmanned camera was positioned to show the counsellor's full face; counselees were seen from behind or from the side. During the visits conducted by counsellors in training, a clinical geneticist was also present. Depending on the available medical information and whether or not a DNA test is conducted, counselees have one or multiple visits. In this paper, the communication process during the initial visit is studied, as for a number of counselees it is the only encounter with the counsellor and for all, during this consultation the foundation for their relationship is laid.

### Measures

The questionnaire contained items assessing counselees' gender, age, education, type of cancer for which they were seeking counselling, and number of affected first- and second-degree relatives. Information on counselee disease status and total number of counselling visits was collected from their medical file. At the start of their participation in the study, the counsellors' gender, age, and profession were assessed.

#### Measurement of preferences: QUOTE-gene^ca^

As described elsewhere (Pieterse *et al*, 2005), a counselee-centred instrument was developed for measuring importance attached to previsit needs and preferences. It contains 25 generic items that relate to what a counselee expects a counsellor to do during counselling, and 19 cancer-specific items that relate to receiving explanations on hereditary cancer in particular. Response options to the individual items were 1 ‘not important’ to 4 ‘extremely important’. Using principal component analysis with varimax rotation, four generic needs were identified: (a) *procedural aspects of counselling* (eight items, Cronbach's *α*=0.84), including what to expect from counselling, (b) *counsellor's sensitive communication* (eight items, *α*=0.84), including being taken seriously and be listened to carefully, (c) *emotional support* (five items, *α*=0.81), and (d) *assessment of susceptibility to the disease* (three items, *α*=0.63) (one item was left out as it loaded equally on two dimensions). Similarly, four cancer-specific needs were identified: (a) *determination and meaning of carrying a cancer gene* (seven items, *α*=0.82), mainly relating to possibilities and limitations of DNA testing, (b) *(emotional) aspects of counselling for counselee and family* (five items, *α*=0.76), (c) *counselee's own risk of developing cancer* (three items, *α*=0.80), and (d) *heredity of cancer in general* (three items, *α*=0.76) (one item was left out as it loaded below 0.40 on any dimension). Importance scores on each issue were computed as the mean score on the relevant items (range score 1–4).

### Coding instruments

Communicative behaviours were rated through the use of an adapted version of the Roter Interaction Analysis System (RIAS), a widely used observation system for coding both physician and patient communication (Roter and Larson, 2002). The system distinguishes mutually exclusive and exhaustive categories into which verbal utterances that convey a complete thought can be classified. A distinction is made between affective or socio-emotional categories, and instrumental or task-oriented categories (Appendix A1). Affective categories carry explicit emotional content and refer to aspects of communication that are needed to establish a therapeutically effective relationship. Task-oriented categories refer to utterances that primarily focus on addressing patients' biomedical or psychosocial problems. Coded elements can be used in analyses separately, or can be grouped into larger conceptual composites. The adaptation of the coding system in this study consisted of the elaboration of categories for coding the exchange of pedigree data, medical information about relatives, communication within the family, and agenda, that is, all knowledge and questions that delineate what the interactants bring to the encounter and/or express they had planned to discuss during the visit.

Additionally, the following three measures were assessed: *length of the visit*; counselees' and counsellors' *contribution* to the encounter relative to the total count of utterances, as a measure of participation in the consultation; and counselees' and counsellors' *psychosocial focus*, that is, the ratio of psychosocial/biomedical questions and information (and education, for counsellors).

Moreover, a checklist was designed to assess whether or not 11 specific topics were discussed during the visit, and if it was the counsellor's initiative. Topics assessed were selected by expert opinion for their relevance. Topics believed to be standard to counselling were excluded, such as pedigree and (family) history of cancer. Topics related to reasons for encounter, predisposition to cancer, and DNA testing.

### Coding reliability

Four coders were trained. The main coder coded 101 (54%) of all consultations. Intra- and intercoder reliability were computed for counsellor and counselee categories with mean occurrence greater than 2% (Roter *et al*, 1991) and proved to be adequate. The main coder recoded a random 10% of her own consultations and reliability coefficients (Pearson's *r*) for counsellor categories averaged 0.81 (range, 0.54–0.96) and counselee categories averaged 0.91 (range, 0.75–0.97). The other coders recoded a random 10% of the main coder's consultations. Intercoder reliability for counsellor categories averaged 0.78 (range, 0.54–0.98) and counselee categories averaged 0.83 (range, 0.59–0.97). Average intercoder reliability over the topics (Cohen's *κ*) was 0.69.

### Statistical analyses

Frequencies of discussing and initiating topics were calculated and whether these varied according to indication for DNA testing and course of counselling (in total one *vs* multiple visits) was tested using *χ*^2^ or Fisher's exact tests, as appropriate.

For analysing the influence of previsit needs on communication behaviours, frequencies of counsellor and counselee utterances were calculated. If two counsellors were present, their utterances were added. Utterances of individuals accompanying counselees are shown, yet were further left out of the analyses as the focus of this paper was on counselees and their previsit needs. Verbal coding categories, where relevant, were combined on the basis of their content (Appendix A1) in line with the hypotheses. As several encounters per counsellor were videotaped, to take account of the resemblance among encounters for one counsellor, multilevel regression analysis was used.

Multilevel regression analyses were carried out for counsellor and counselee communication categories separately and in two steps. First, background variables at counselee level (gender, age, education, and personal and family history of cancer) and counsellor level (gender, age, and profession) were included. Where two participants were seen together by a counsellor, counselee data were used for one (randomly selected) counselee only. If two counsellors were present, characteristics of the counsellor with the largest amount of utterances during the visit were used. The categories ‘clinical geneticist’ and ‘resident’ were aggregated into ‘doctor’ as opposed to ‘nurse’. Only significant background correlates were retained. Second, (all) the previsit need(s) was included in the model as hypothesised (Table 1). In the Results section, only data on significant associations will be presented.

Predictors at interval level measurement were recoded to standardised *z*-scores. If 25% or less of the values were missing on the QUOTE-gene^ca^ subscales, missing values were replaced by the mean. Significance of the regression coefficients was tested using *χ*^2^ tests. Analyses were carried out using SPSS 11.5 and MLwiN 1.10. Significance testing was carried out two-sided at *α*=0.05 level.

## RESULTS

### Participants

#### Counselees

**Figure 1:**
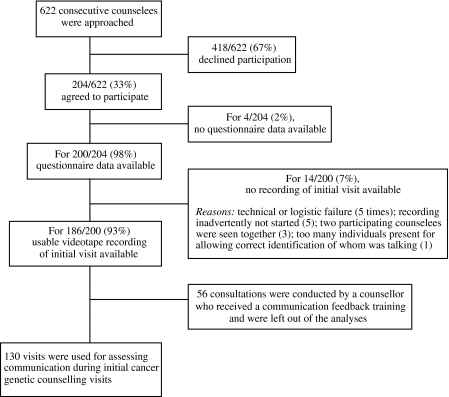
Details of inclusion of counselees.

Data at baseline and a videotape recording of the initial visit were available for analysis for 130 counselees (Figure 1).

In Table 2, relevant counselee characteristics are listed. The counselees with a family history of cancer had one to four (M=1.5, s.d.=0.8) first-degree and one to eight (M=2.1, s.d.=1.5) second-degree affected relatives. Participants were, on average, 2.1 years older than decliners (*P*=0.022) (Pieterse *et al*, 2005); however they did not differ in gender, referral pathway, type of cancer, or (family) history of cancer.

#### Counsellors

Five clinical geneticists (four female, one male), four residents in clinical genetics (three female, one male), and five genetic nurses (all female) participated. Counsellors were aged 29–46 years (M=38.1, s.d.=5.3).

#### Consultations

Consultations lasted 43.9 min (s.d.=13.7) on average. The counsellors conducted 93 visits out of 130 (72%) alone. During the 37 visits out of 130 that were conducted by a clinical geneticist together with a resident or a nurse, the counsellor with the largest amount of utterances averaged 468.4 statements (s.d.=162.9; min.=199, max.=821), compared to a mean of 138.5 utterances by the other counsellor (s.d.=85.5; min.=6, max.=369). Moreover, in 29 (78%) of these 37 visits, the former spoke at least two times more than the latter. In 63 of the 130 (48%) visits, counselees were seen alone. During all other consultations, counselees were accompanied by one (*N*=61) or two or more individuals (*N*=6). Utterances of companions added up to 6.7% of the total communication (Table 3).

### Counselee and counsellor conversational contribution

On average, 19% of counselee and 14% of counsellor communication related to socio-emotional communication, mainly (16.8. *vs* 11.4%) indications of acknowledgment or agreement (Table 3).

Task-oriented communication consisted largely of counselees providing information (17.5%) and counsellors informing and educating counselees (21.0%). The counselees mainly provided information on their family history of cancer, medical condition, and pedigree, adding up to 26% of counselee talk (not in the table). They further essentially provided information on their agenda and on psychosocial issues, each category adding up to 6% of counselee talk. The counsellors asked counselees about their agenda in 123 of the 130 visits and the counselees provided information on their agenda in 129 of the 130 visits. The counsellors mostly provided information on general or counselee-related medical issues, as well as medical advices for counselees and their relatives, utterances that amounted to 33% of counsellor talk (not in the table).

The counselees contributed significantly less to the interaction than the counsellors, a mean of 40 *vs* 53% utterances (paired *t*=11.8, *P*=0.000). In particular, counsellors asked significantly more questions (3.7 *vs* 0.6%; paired *t*=17.45, *P*=0.000) and made significantly more orienting or directive remarks (5.8 *vs* 1.0%; paired *t*=19.84, *P*=0.000). The counsellors also used significantly more paraphrases (7.9 *vs* 2.1%; paired *t*=19.47, *P*=0.000), thereby confirming shared understanding and knowledge. This included summaries of family history of cancer, information that the counsellor gathered from the counselee prior to or during the visit. The counselees had a significantly stronger psychosocial focus than counsellors (paired *t*=−10.57, *P*=0.000).

### Topics covered during the visit

Overall, the counselees' motives for and referral pathway to counselling were almost always a topic of discussion, in contrast to their risk perceptions (Table 4).

Also, predisposition to cancer and possibilities, limitations, and procedure of DNA testing were discussed in a majority of visits. Medical consequences of testing were discussed in more than half and emotional consequences in less than half of the visits. In 79–99% of the visits, the counsellor initiated the discussion of a topic, except for emotional consequences of DNA testing, where counsellors took the initiative in just over half (31 out of 53; 59%) of the visits in which it was discussed (not in the table).

Indication for DNA testing and whether the initial visit would be the only one or not did not affect the frequency of discussing reasons for encounter. With counselees for whom DNA testing was indicated, mode of inheritance and meaning of genetic predisposition were discussed significantly more often compared to those without an indication (*P*=0.015 and 0.040, respectively), as well as possibilities (*P*=0.001), procedure (*P*=0.000), and medical consequences of testing (*P*=0.043). Compared to those who would be seen again, how often the type of cancer is genetic and procedure of DNA testing were discussed less often with counselees for whom the initial visit was the only visit (*P*=0.009 and 0.001, respectively).

### Influence of previsit needs on counselee communication

Previsit needs slightly affected counselee communication in that there was a trend for counselees considering it more important to receive explanations on the assessment of cancer susceptibility to ask more medical questions (*B*=0.20, *χ*^2^=3.74, *P*=0.053) and those attaching more weight to be taken seriously and be listened to carefully, to provide more information on their agenda (*B*=0.14, *χ*^2^=2.98, *P*=0.084).

### Influence of previsit needs on counsellor communication

The counselees' previsit needs affected counsellor communication in that counsellors provided more psychosocial information and education to counselees who had higher previsit needs for emotional support (*B*=0.21, *χ*^2^=4.29, *P*=0.038).

## DISCUSSION

In the present study, a detailed analysis of the interaction during the initial cancer genetic counselling visit was carried out, and the extent to which counselees' previsit needs as measured with the QUOTE-gene^ca^ are reflected in the communication was assessed. It revealed that counselees' previsit needs only slightly affected counselee and counsellor communication behaviours.

### Course and content of the interaction

The results show that on average counsellors contribute more to the interaction than do counselees, in agreement with Butow and Lobb (2004). The proportion of socio-emotional talk was analogous to other secondary care settings (15% for specialist and 21% for patient) (Van Dulmen, 1999). Counsellors directed the communication more than did counselees, partly by using orienting utterances, a structuring behaviour that is seen as part of basic medical tasks (Roter, 2000). They also asked more questions than counselees, and these concerned three times more often biomedical than psychosocial issues. This finding is not surprising, as primarily the likelihood that cancer running in the family is genetic needs to be estimated, and that counselees do not always provide sufficient (family) medical and pedigree information prior to the encounter. The counsellors also directed the interaction more by initiating the discussion of the medical topics assessed, in a large majority of the visits.

In almost all visits, the counselees' agenda was discussed, and counsellors asked about it in a higher proportion of visits than Butow and Lobb (2004) found (i.e., in 123 out of 130 or 95% compared to 69%). It is unknown in how far counselees' agenda was followed. The counselees were found to ask relatively few questions compared to counsellors and to spend only a mean of 0.6/40.4, that is, 2% of their utterances, in asking questions (Table 3). As Butow and Lobb (2004) noted, passive listening reduces understanding and especially where substantial amounts of new information are presented, a more interactive discussion might increase counselees' understanding and recall. Low patient question-asking behaviour was noted by various authors in other medical settings (Beisecker and Beisecker, 1990; Street, 1991; Ford *et al*, 1996). As patient question-asking was found to be strongly related to physicians' provision of medical information (Street, 1991; Butow *et al*, 1995), counselees might moreover elicit more effective responses from counsellors if they would make more use of this directing tool. Low question-asking may be related to counselee concession to expert authority, or to unfamiliarity with the content and process of cancer genetic counselling (Collins *et al*, 2000), or the exact role of counsellors in the provision of health care (Bernhardt *et al*, 2000). Hallowell *et al* (1997) found that unfamiliarity with cancer genetic counselling inhibited counselees to formulate questions in advance, and that a large minority envisaged their role as passive. Limited question-asking behaviour might also result from counselees having gathered ample information about hereditary cancer, screening, and risk management before their visit. The information counsellors subsequently provide may then be relatively easy to understand. However, results showing counselees' disappointment about the amount of information received during counselling (Hallowell *et al*, 1997) and high expectations of having a DNA test carried out (Pieterse *et al*, 2005) while the availability of this option is limited, contradict this proposition. It is conceivable that the large amount of information conveyed during the visit at the counsellors' initiative may not only have answered at least partly counselees' questions, but may also require time for counselees to sink in.

Moreover, the interaction was mainly focused around biomedical issues. It is remarkable that potential emotional consequences of DNA testing were discussed with less than half of counselees who had an indication for testing, for themselves or a relative. Even counselees who have thought through the option and clearly state they want a test might overlook how this decision or the test result may emotionally disturb themselves or their family. It would seem appropriate to discuss this topic with counselees who have an indication, before DNA testing is started. Counselees had a stronger psychosocial focus than counsellors, as was also illustrated by them initiating more often the discussion of emotional consequences of DNA testing, compared to the other topics assessed. Apparently, psychosocial issues are rather on counselees' than on counsellors' agenda. Alternatively, counselees may have felt invited to bring forward socio-emotional issues, for instance by counsellors' eye gaze. This was found to be a powerful instrument for doctors to detect potential psychosocial problems in patients (Bensing *et al*, 1995). *Post hoc* testing showed that the amount of counsellor eye gaze was significantly related to the amount of psychosocial information counselees provided.

Compared to the other topics assessed, risk perception appears not to be a major subject during the initial visit. Where counselling is continued, actual risk may often not yet be clear at this stage; thus, discussing risk perceptions may be postponed. However, even with counselees seen at the clinic only once, their risk perceptions were not examined against actual risks as could be best established at the time. Studies showed that after counselling, risk perceptions were reduced compared to baseline; nonetheless, inaccuracies persisted (Cull *et al*, 1999; Collins *et al*, 2000; Lobb *et al*, 2003). Probing these perceptions and discussing them where they greatly deviate from the counsellor's estimation may improve accuracy. Topics relating to predisposition to cancer and DNA testing were increasingly often discussed with counselees for whom it appears more relevant. However, all medical topics except procedure and medical consequences of testing were discussed with two-thirds or more of all counselees, exemplifying the large amount of medical information that is conveyed nearly standard during counselling.

### Influence of previsit needs on communication

Results suggest that counselees' previsit needs minimally influence the interaction during the initial visit and counselees did not appear to tailor their verbal communication to their previsit needs. This apparent reluctance to explicitly ask for desired information (Roter, 1977; Street, 1991) or to express directly emotional concerns (Street, 1991; Suchman *et al*, 1997), was noted earlier in primary care patients.

Counsellors appeared sensitive to counselees' needs for emotional support, although not by questioning them more about their concerns, replicating the finding by Butow and Lobb (2004). Counselees may be reluctant to mention emotional issues, due to psychological embarrassment or hesitation to trouble the counsellor, or possibly because they feel deterred by counsellor's interview behaviours as was found in primary care (Cape and McCulloch, 1999). It may be advisable to counsellors to invite the discussion of psychosocial issues so as to assess whether counselees have questions or whether they desire to share their concerns, and to be able to respond more adequately to counselee-specific issues.

### Limitations

First, the response rate of counselees was low. It may have been due to procedural requirements for indicating interest, as increased study requirements (Helmes *et al*, 2000; Friebel *et al*, 2004) and understanding written information (Cox, 2002; Friebel *et al*, 2004) may discourage participation. Furthermore, as the counsellor was unknown to eligible counselees at the time and was not involved in the recruitment, low participation may also have resulted from the lack of a ‘trusted source’ that explicitly recommended participation (Kreiger *et al*, 2001). Additionally, videotaping may have adversely affected willingness to participate. Findings from Howe (1997) suggest that patient refusal to have their primary care consultation videotaped may be associated with complex contextual and socio-demographic factors, and with decreasing age in particular. In our study, participants indeed were older than decliners; however, they did not differ on other background measures (Pieterse *et al*, 2005). It is unknown whether participants and decliners differed on their needs, and thus how representative our sample is for the larger population. Also, 94% of participating counselees were female, half (55%) of the counselees were higher educated, and a majority (64%) sought counselling for hereditary breast cancer. The results should therefore only be generalised with caution to men, to the broader population of, on average, lower educated women at increased risk of developing hereditary cancer, and to other types of hereditary cancers than breast cancer.

Second, we did not record counsellor–counselee phone or mail contacts, so we cannot exclude with certainty that topics that were not discussed during initial visits at the clinic were actually not discussed with counselees who were seen once only.

Third, a stronger association between verbal communication and counselee previsit needs may be found using an interaction coding instrument that is specifically designed to code for the needs that were measured using the QUOTE-gene^ca^.

## CONCLUSION

Counsellors appear to dominate initial cancer genetic counselling visits, while counselees play a more passive role in the interaction than one might expect, given that genetic counselling is rather a medical possibility than a necessity. Results suggest counselees to have a stronger psychosocial agenda compared to counsellors; however, they may not be sufficiently acquainted with this specialist health service to come well prepared and take a more active role in the encounter. Counsellors appear to tailor their communication to counselees' emotional needs, however not by enquiring more about psychosocial issues or showing more empathy. Neither do counsellors adapt their communication to counselees' informational previsit needs. Yet, counselees do not appear to readily verbalise their prior needs. Newly referred counselees may be helped by receiving more information on the counselling procedure prior to the consultation, and they may be advised on how to prepare their visit. They may, for example, be encouraged to write down questions in advance, possibly helped by a prompt sheet such as was successfully devised for promoting cancer patients' participation (Brown *et al*, 1999; Wells *et al*, 2004), or to send questions ahead to the counsellor.

## Figures and Tables

**Table 1:** Hypothesised positive associations between counselee (CE) and counsellor (CR) communication, and CE previsit need(s)

**Communication behaviour**	**CE previsit need**
*Counselee*	
Expression of concern	Emotional support
Medical questions	Explanations on procedural aspects of counselling
	Explanations on the assessment of cancer susceptibility
	Explanations on the determination/meaning of carrying a cancer gene
	Explanations on CE own risk of cancer
	Explanations on heredity of cancer
Psychosocial questions	Explanations on (emotional) aspects of counselling for CE/relatives
Psychosocial information	Emotional support
Information on agenda	CR sensitive communication
	
*Counsellor*	
Expression of empathy	Emotional support
Medical information/education	Explanations on procedural aspects of counselling
	Explanations on the assessment of cancer susceptibility
	Explanations on the determination/meaning of carrying a cancer gene
	Explanations on CE own risk of cancer
	Explanations on heredity of cancer
Psychosocial questions	Emotional support
Psychosocial information/education	Emotional support
	Explanations on (emotional) aspects of counselling for CE/relatives
Education on agenda	Explanations on procedural aspects of counselling

**Table 2:** Counselee demographics, history of cancer, and course of counselling

	***N***	**%**
*Age (years)*		
Mean (s.d.)	44.5 (9.4)	
Range	18–72	
		
*Gender*		
Female	122	94
Male	8	6
		
*Education*		
High school level	58	45
Secondary level	72	55
		
*Type of cancer*		
Breast cancer	83	64
Colon cancer	25	19
Breast and colon cancer	9	7
Ovarian cancer	8	6
Other cancers	5	4
		
*Personal history of cancer*		
Counselee affected with cancer	63	49
Counselee unaffected with cancer	67	52
		
*Family history of cancer*		
First- or second-degree relatives affected with cancer	114	91
No first- or second-degree relatives affected with cancer	12	10
		
*Indication for DNA testing* a		
Yes	86	66
No	21	16
Unclear^b^	23	18
		
*Total number of visits*		
1	44	34
2 or more	86	66

Summations vary because of missing data.

a Indication for testing the counselee or a relative as judged after the initial visit.

b Indication for DNA testing unclear due to missing medical information.

**Table 3:** Mean and percentage^a^ of occurrence of the various coding categories (*N*=130 visits)

	**Counsellor(s)**			**Counselee**			**Other(s)^b^**		
	**M**	**s.d.**	**%**	**M**	**s.d.**	**%**	**M**	**s.d.**	**%**
**Socio-emotional communication**									
Social talk	22.6	19.2	2.1	18.8	12.9	1.7	6.5	14.7	0.5
Agreement	128.4	64.7	11.4	183.8	88.8	16.8	30.2	50.3	2.5
Concern	0.1	0.5	0.0	0.8	1.4	0.1	0.1	0.6	0.0
Verbal attention	3.7	3.4	0.4	—	—	—	—	—	—
Reassurance	1.2	2.0	0.1	0.1	0.4	0.0	0.0	0.2	0.0
Disagreement	1.1	1.6	0.1	3.0	2.9	0.3	0.6	1.5	0.0
*Total*	157.1	69.7	14.1	206.5	92.4	18.9	37.5	62.1	3.0
									
**Task-oriented communication**									
Orientation	60.1	31.4	5.8	10.3	7.6	1.0	2.4	5.7	0.2
									
*Partnership building behaviour*									
Clarification	1.7	3.2	0.2	0.6	0.9	0.1	0.1	0.5	0.0
Paraphrase	83.2	33.2	7.9	23.8	19.7	2.1	6.4	10.4	0.6
Opinion	1.4	11.0	0.1	0.1	0.3	0.0	0.0	0.1	0.0
*Total*	86.3	35.6	8.2	24.4	19.8	2.2	6.5	10.7	0.6
									
*Questions*									
Medical condition, counselee	3.6	3.6	0.3	4.7	5.5	0.4	1.6	3.2	0.1
Screening/prophylactic surgery, counselee	1.2	2.1	0.1	0.6	1.4	0.1	0.1	0.5	0.0
Medical condition, relative(s)	16.6	10.5	1.6	1.0	1.4	0.1	0.4	1.1	0.0
Pedigree data	7.9	7.6	0.8	0.2	0.6	0.0	0.1	0.7	0.0
Communication within family	1.0	1.4	0.1	0.1	0.3	0.0	0.0	0.1	0.0
Lifestyle	0.6	1.4	0.1	0.1	0.3	0.0	0.0	0.2	0.0
Agenda	3.9	2.7	0.4	0.0	0.0	0.0	0.1	0.4	0.0
Psychosocial issues	2.4	2.4	0.2	0.1	0.4	0.0	0.1	0.4	0.0
Current feelings	0.0	0.2	0.0	0.2	0.6	0.0	0.0	0.0	0.0
*Total*	37.2	18.7	3.7	6.9	7.1	0.6	2.5	4.7	0.2
									
*Information*									
Medical condition, counselee	139.2	68.7	12.8	27.7	28.2	2.4	4.1	9.6	0.3
Screening/prophylactic surgery, counselee	9.4	13.4	0.8	6.1	7.8	0.5	0.6	2.0	0.0
Medical condition, relative(s)	6.9	8.1	0.7	62.7	38.1	6.0	11.3	24.8	1.0
Pedigree data	0.7	1.3	0.1	22.7	17.0	2.2	3.6	9.7	0.3
Communication within family	0.4	0.8	0.0	7.3	6.8	0.7	0.8	2.1	0.1
Lifestyle	0.6	1.8	0.1	7.2	10.8	0.6	1.1	3.1	0.1
Agenda	3.0	2.9	0.3	26.2	16.9	2.4	3.7	8.4	0.3
Psychosocial issues	3.1	5.0	0.3	30.4	28.2	2.6	5.3	11.9	0.4
Current feelings	0.2	0.5	0.0	1.5	2.3	0.1	0.3	2.1	0.0
*Total*	163.6	78.1	15.1	191.8	90.1	17.5	30.8	58.7	2.5
									
*Counselling*									
Cancer risk/DNA test, counselee	30.3	20.8	2.9	—	—	—	—	—	—
Screening/prophylactic surgery, counselee	7.2	7.7	0.7	—	—	—	—	—	—
Cancer risk/screening advice, relative(s)	18.3	18.3	1.6	—	—	—	—	—	—
Advice/request about family communication	1.7	2.7	0.2	—	—	—	—	—	—
Lifestyle	0.0	0.0	0.0	—	—	—	—	—	—
Education about agenda	0.3	0.9	0.0	—	—	—	—	—	—
Education about psychosocial issues	6.6	9.6	0.6	—	—	—	—	—	—
*Total*	64.5	34.7	5.9	—	—	—	—	—	—
									
Other utterances	1.1	1.7	0.1	1.9	2.5	0.2	1.2	2.6	0.1
									
**Additional measures**									
Psychosocial focus	0.0	0.0		0.3	0.3				—
Conversational contribution			52.9			40.5			6.7

Summations may vary due to rounding off.

a Percentages were calculated with regard to the total amount of utterances of *all* participants in the encounter.

b Individuals accompanying counselees; their utterances were not added to counselees' in the analyses.

**Table 4:** Frequencies (%) of discussing topics during the initial visit

		**Indication for DNA test^a^**		**Total number of visits**	
**Topic**	**Overall (*N*=130)**	**No (*N*=21)**	**Yes (*N*=86)**	**1 (*N*=44)**	**2 or more (*N*=86)**
*Reason for encounter*					
Motive for counselling	93.0	90.0	94.2	93.0	93.0
Referral pathway	88.3	90.5	86.9	90.7	87.1
Counselee risk perceptions	14.6	23.8	15.1	13.6	15.1
					
*Predisposition to cancer*					
Mode of inheritance	80.0	66.7	89.5*	72.7	83.7
Prevalence of hereditary type of cancer	80.8	76.2	83.7	68.2	87.2**
Meaning of genetic predisposition	77.5	66.7	88.2*	69.8	81.4
					
*DNA testing*					
Possibilities	88.4	71.4	97.7**	86.0	89.5
Limitations	82.3	76.2	89.5	84.1	81.4
Procedure	76.0	23.8	94.2***	58.1	84.9**
Medical consequences	60.8	47.6	70.9*	52.3	65.1
Emotional consequences	40.8	28.6	48.8	38.6	41.9

a Totals do not add up to 130 because indication for DNA testing was unclear after the initial visit for 23 out of 130 counselees.

* *P*<0.05;

** *P*<0.01;

*** *P*<0.001.

**Table A1:** Counselee (CE) and counsellor (CR) individual coding categories and combinations of these used in the analyses

*Socio-emotional communication*	
Social talk	Personal remarks, social conversation, laughs, jokes, approval, compliments
Agreement	Signs of acknowledgement, agreement or understanding
Concern^a^	Immediate emotional or psychosocial worries, crying
Verbal attention^a^^,^^b^	Legitimise, empathy, partnership, support
Reassurance^a^	Give reassurance, ask for reassurance
Disagreement	Disapproval, criticism, disagree
	
*Task-oriented communication*	
Orientation	Directive remarks regarding the course of the visit, division of roles between counsellors
Clarification	Bid for repetition, ask for understanding
Opinion	Ask for opinion, give opinion
Paraphrase	Checks for understanding, confirm shared understanding or knowledge
Medical questions^c^	Questions that ask for information on medical condition or screening or prophylactic surgery
Nonmedical questions	Questions that ask for information on pedigree, communication within family, lifestyle, agenda, psychosocial issues,^d^ or current feelings^d^
Medical information^e^	Statements or facts relating to medical condition or screening or prophylactic surgery
Nonmedical information	Statements or facts relating to pedigree, communication within family, lifestyle, agenda, psychosocial issues,^f^^,^^g^ or current feelings^f^
Medical education^b^^,^^e^	Statements relating to risk of cancer or indication for DNA testing, or statements that suggest action to be taken by the other relating to screening or prophylactic surgery
Psychosocial education^b^	Statements which suggest resolution or action to be taken by the other relating to family communication, lifestyle, or psychosocial issues,^g^ or statements aimed at education about psychosocial issues^g^ or agenda
Other	Other utterances, unintelligible utterances

a Combined into CR empathy.

b CR only.

c General medical information and medical information concerning CEs specifically were distinguished from medical information relating to CEs' relative(s).

d Combined into CR psychosocial questions.

e Combined into CR medical information and education.

f Combined into CE psychosocial information.

g Combined into CR psychosocial information and education.

## Appendix A1

Counselee (CE) and counsellor (CR) individual coding categories and combinations of these used in the analyses are given in Table A1.
